# Predictors for failure of supraglottic superimposed high‐frequency jet ventilation during upper airway surgery in adult patients; a retrospective cohort study of 224 cases

**DOI:** 10.1111/coa.13465

**Published:** 2019-12-12

**Authors:** Gyorgy B. Halmos, Charlotte M. A. Plate, Grita Krenz, Bouwe Molenbuur, Frederik G. Dikkers, Boukje A. C. van Dijk, Jan E. Wachters

**Affiliations:** ^1^ Department of Otorhinolaryngology/Head and Neck Surgery University Medical Center Groningen University of Groningen Groningen The Netherlands; ^2^ Department of Anaesthesiology University Medical Center Groningen University of Groningen The Netherlands; ^3^ Department of Otorhinolaryngology Amsterdam University Medical Center University of Amsterdam The Netherlands; ^4^ Department of Research Netherlands Comprehensive Cancer Organisation (IKNL) Utrecht The Netherlands; ^5^ Department of Epidemiology University Medical Center Groningen University of Groningen Groningen The Netherlands

**Keywords:** endoscopic upper airway surgery, obesity, predictor, pulmonary disease, supraglottic superimposed high frequency jet ventilation

**Key points**

- Supraglottic superimposed high‐frequency jet ventilation (SSHFJV) maximises surgical field during endoscopic upper airway surgery.
- In our retrospective series of 224 cases, there was a low incidence (12%) of failure with the use of SSHFJV in upper airway surgery.
- Positive history of pulmonary pathology (OR = 4.91) and high BMI (OR = 1.15) were found to be significant independent factors for failure of SSHFJV in adult patients undergoing upper airway surgery.
- Converting ventilation techniques could be safely performed when SSHFJV failed.
- SSHFJV is a safe ventilation technique during upper airway surgery, even in combination with the application of CO_2_ laser.

## 1 INTRODUCTION

During endoscopic upper airway surgery, anaesthetists and surgeons have to share the airway. Therefore, alternative ventilation techniques have been developed in the past decades. To optimise the surgical field high‐frequency jet ventilation (HFJV) was developed. A “tubeless” HFJV method has been introduced in the late 90s: supraglottic superimposed HFJV (SSHFJV).^1^ Other, frequently used tubeless technique is spontaneous breathing with propofol‐remifentanil anaesthesia with or without high‐flow nasal oxygenation.^2^, ^3^ During SSHFJV, surgery is performed through a laryngoscope which has integrated jet stream nozzles enabling ventilation and no catheter is needed, in contrast to conventional HFJV. Using SSHFJV, there is completely free access of the surgical field and adequate oxygenation and ventilation can be achieved during surgery. SSHFJV also lowers the chance of airway burn during laser surgery, as no flammable tube or catheter is needed. As no disposables (like catheters in conventional HFJV) are used during SSHFJV, it seems to be a cheaper technique; however, a cost‐effectiveness study has not been performed yet. The only disadvantage of SSHFJV seems to be obligatory visualisation of the airway through the ventilating laryngoscope during the whole procedure, otherwise the ventilation of the patient is not possible, which makes it not suitable, for instance, for intervention in the hypopharynx. According to previous reports, SSHFJV is a safe ventilation method, even in patients with severe cardiovascular and pulmonary comorbidities.^4^, ^5^ However, sometimes ventilation has to be temporarily or definitively converted into endotracheal tube ventilation because of drop in O_2_ saturation and accumulation of CO_2_.^6^

The aim of the present study was to identify factors which can predict failure of SSHFJV in upper airway surgery.

## 2 MATERIALS AND METHODS

### 2.1 Ethical considerations

Data were retrospectively collected and the anonymity of the patients has been guaranteed; therefore, no approval of the Institutional Review Board is needed in accordance with Dutch Medical Research Law legislation.

### 2.2 Patients

This retrospective study included 163 adult patients who underwent 224 upper airway procedures with SSHFJV between November 2007 and November 2017 at our tertiary referral centre.

### 2.3 Supraglottic superimposed high‐frequency jet ventilation

**Figure 1:**
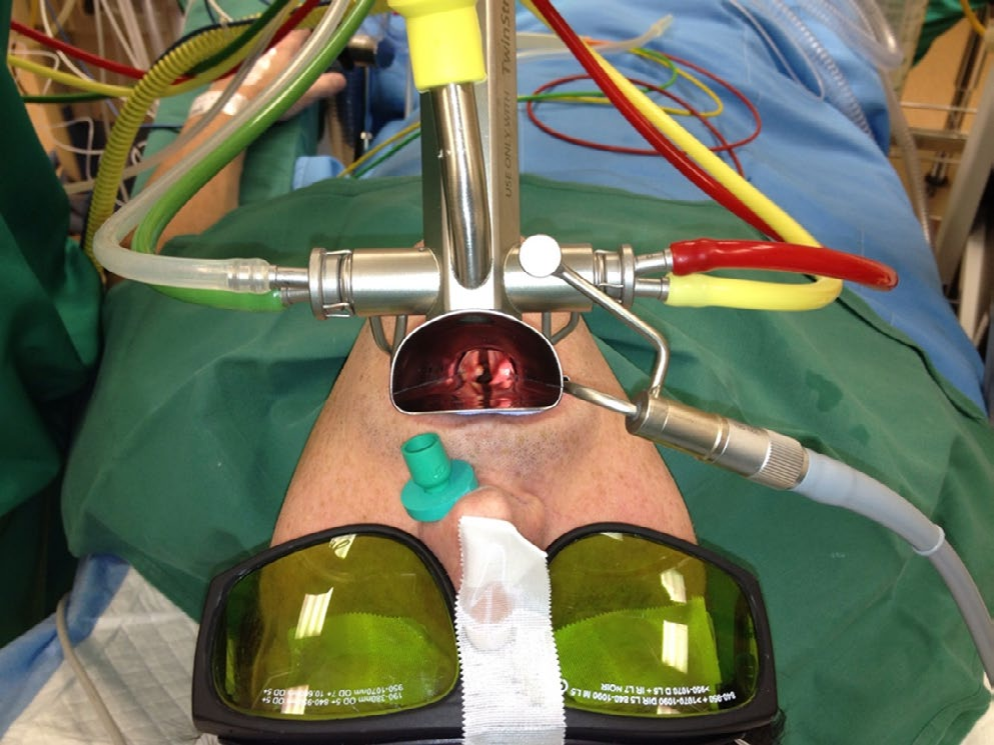
Setup of a patient during transoral microsurgical procedure using CO_2_ laser, ventilated with SSHFJV. Note the unhampered sight of the complete glottis with an exophytic lesion on the left vocal process. Green and colourless tube on left side: high‐ and low‐frequency airflow. Yellow tube in centre: humidification and heating of entrained air. Red and yellow tube on right side: continuous measurement of O_2_ and CO_2_ levels distally in modified Bouchayer laryngoscope. Note the green nasopharyngeal tube in the left nostril yielding unhampered additionally entrained transnasal airflow

Under general anaesthesia, after pre‐oxygenation through a mask, a modified laryngoscope (Jet Laryngoscope; Carl Reiner GmbH) was inserted and the SSHFJV was connected (TwinStream™ Multi Mode Respirator; Carl Reiner GmbH). (Figure 1) During SSHFJV, two jet streams with different frequencies are being used at the same time. One jet stream fires at a high frequency and is continuous, the low frequency is biphasic, providing an inspiratory and expiratory phase.

### 2.4 Variables

Relevant data from the electronic patients’ files were extracted and retrospectively analysed. Clinical imaging data were available in all cases and were reassessed to estimate the severity of the airway stenosis.

The following variables were extracted from the electronic patients’ files: age, sex, weight, smoking status, comorbidity status (according to the Adult Comorbidity Index‐27 (ACE‐27) and the American Society of Anaesthesiologist Physical Status (ASA), and the presence of cardiovascular or pulmonary pathology), body mass index (BMI), airway anatomy (Cormack‐Lehane grade and Mallampati score), level of the actual airway pathology (supraglottic, glottic and subglottic), severity of airway stenosis (in percentage and according to the Cotton‐Myer grading scale), diagnosis and type of surgery (including the application of laser).

### 2.5 Outcome measure

When the saturation drops during ventilation with SSHFJV the anaesthesiologist may choose to intubate with endotracheal tube for a short period of time to reoxygenate. When the saturation of the patient is normalised, the operation with SSHFJV can be continued (temporary conversion); however, sometimes it is not possible (definitive conversion). Failure of SSHFJV was defined as temporary and/or definitive conversion.

### 2.6 Statistical analysis

Patient characteristics and other variables were compared between the converted and non‐converted patients using chi‐square test (or Fisher's exact test). The *t* test was used in case of continuous variables.

The potentially predictive variables for conversion of ventilation were analysed using univariable logistic regression. Odds ratios with corresponding 95% confidence intervals and *P*‐values were calculated.

Statistically significantly variables were included in the multivariable logistic regression model and analysed using the backward stepwise method. All statistical analyses were performed using ibm spss statistics 23.0 (IBM, Armonk).

## 3 RESULTS

### 3.1 Patients characteristics

In 198 (88%) cases, satisfactory ventilation by SSHFJV was achieved; however, during 26 interventions (12%) ventilation had to be definitively or temporarily converted to endotracheal tube ventilation. The main reason for conversion was desaturation of the patient (25/26 cases).

A detailed rendering of the patient characteristics, including age, sex, weight, smoking status, laryngological history, anatomical level of the pathology, diagnosis, treatment and comorbidities, are described in Table 1. Of the converted cases, 14 had a history of pulmonary diseases: six had chronic obstructive pulmonary disease (COPD), two had bronchial asthma, three patients suffered from sarcoidosis involving the lungs and two had obstructive sleep apnoea (OSA). The mean BMI was 34 in the converted group, vs 28 in the non‐converted group (*P* = <.001). In the converted group, the percentage of obstruction was estimated at 51% compared with 33% in the non‐converted group (*P* = .011). There was no difference in CO_2_ laser use between conversion and non‐conversion groups (77% and 71%, respectively; *P* = .543)*.* No complications due to use of SSHFJV were observed in any of patients.

**Table 1:** Patient characteristics of the study population divided into converted and non‐converted group

	Non‐converted, N (%)	Converted, N (%)	*P*‐value
N	198 (88%)	26 (12%)	
Age			
Mean ± SD	58 ± 17	61 ± 15	.432†
Median (Range)	60 (19‐90)	64 (20‐83)	
Sex			
Male	103 (52%)	10 (38%)	.194
Female	95 (48%)	16 (62%)	
BMI			
Mean ± SD	28 ± 6	34 ± 5	**<.001**†
Median (Range)	27 (18‐50)	34 (25‐45)	
Missing	1	0	
Weight			
Mean ± SD	82 ± 19	100 ± 22	**<.001**†
Median (Range)	79 (50‐148)	86 (65‐151)	
Missing	2	0	
Smoking status			
Current smoker	61 (31%)	7 (27%)	.517‡
Past smoker	30 (15%)	2 (8%)	
Never smoked	107 (54%)	17 (65%)	
History of cardiovascular pathology			
Yes	78 (39%)	12 (46%)	.509
No	120 (61%)	14 (54%)	
History of pulmonary pathology			
Yes	41 (21%)	14 (54%)	**<.001**
No	157 (79%)	12 (46%)	
ACE‐27 total			
0, 1	125 (63%)	16 (62%)	.874
2, 3	73 (37%)	10 (38%)	
ACE‐27 cardiovascular			
0, 1	174 (88%)	22 (85%)	.544‡
2, 3	24 (12%)	4 (15%)	
ACE‐27 pulmonology			
0, 1	194 (98%)	26 (100%)	1.000‡
2, 3	4 (2%)	0 (0%)	
ASA Class			
1, 2	155 (78%)	14 (56%)	**.014**
3, 4	43 (22%)	11 (44%)	
missing data	0	1	
Laryngological history			
Positive	109 (55%)	16 (62%)	.531
Negative	89 (45%)	10 (38%)	
% Obstruction of the lumen			
Mean ± SD	33 ± 34	51 ± 40	**.011**†
Median (Range)	20 (0‐95)	70 (0‐98)	
Missing	1	0	
Cormack‐lehane			
1, 2	98 (99%)	17 (94%)	.285‡
3, 4	1 (1%)	1(6%)	
Missing data	99	8	
Mallampati			
1, 2	149 (78%)	20 (80%)	.786
3, 4	43 (22%)	5 (20%)	
Missing data	6	1	
Anatomical level of pathology			
Supraglottic	41 (21%)	8 (31%)	.332
Glottic	97 (49%)	9 (35%)	
Subglottic and tracheal	60 (30%)	9 (35%)	
Oncological origin of pathology			
Yes	120 (61%)	13 (50%)	.301
No	78 (39%)	13 (50%)	
Treatment			
Intervention on airway stenosis	45 (23%)	3 (12%)	.480‡
Excision (pre)malignant	40 (20%)	5 (19%)	
Debulking tumour	37 (19%)	7 (27%)	
Excision benign lesion	49 (25%)	9 (35%)	
Other, diagnostic procedure	27 (14%)	2 (8%)	
Use of laser			
Yes	141 (71%)	20 (77%)	.543
No	57 (29%)	6 (23%)	

Note

Statistical test used: chi‐square test, Fisher's exact test (marked with ‡) or with *t* test (marked with †). Significant *P*‐values are indicated with bold numbers.

Abbreviations: ACE‐27, Adult Comorbidity Evaluation‐27 index; ASA Class, American Society of Anaesthesiologist Physical Status Classification; BMI, body mass index; SD, standard deviation.

### 3.2 Univariable analysis

In univariable analyses (Table 2), we found a statistically significant higher risk of conversion for increasing BMI (OR = 1.15; 95% CI: 1.08‐1.22), for a positive history of pulmonary pathology (OR = 4.47; 95% CI: 1.92‐10.39), a higher ASA Class (3‐4 vs 1‐2) (OR = 2.40; 95% CI: 1.20‐6.69), and for a higher percentage of obstruction (OR = 1.02; 95% CI: 1.00‐1.03).

**Table 2:** Univariable logistic regression analysis of patient and surgical factors contributing to SSHFJV failure

	OR (95% CI)	*P*‐value
Age	1.01 (0.99‐1.04)	.431
Sex		
Male	1	
Female	1.74 (0.75‐4.01)	.198
BMI	1.15 (1.08‐1.22)	**<.001**
Smoking status		
Current smoker	1	
Past smoker	0.58 (0.11‐2.97)	.514
Never smoker	1.39 (0.54‐3.53)	.495
History of cardiovascular pathology		
Yes	1.32 (0.58‐3.00)	
No	1	.510
History of pulmonary pathology		
Yes	4.47 (1.92‐10.39)	
No	1	**.001**
ACE‐27 total		
0, 1	1	
2, 3	1.07 (0.46‐2.48)	.874
ACE‐27 cardiovascular		
0, 1	1	
2 ,3	1.32 (0.42‐4.15)	.637
ASA Class		
1, 2	1	
3, 4	2.40 (1.20‐6.69)	**.018**
Laryngological history		
Positive	1	
Negative	0.77 (0.33‐1.77)	.532
% Obstruction of the lumen	1.02 (1.003‐1.03)	**.013**
Mallampati		
1, 2	1	
3, 4	0.79 (0.31‐2.44)	.786
Anatomical level of pathology		
Supraglottic	1	
Glottic	0.48 (0.17‐1.32)	.153
Subglottic, Tracheal	0.77 (0.27‐2.16)	.617
Oncological origin of pathology		
Yes	1	
No	0.65 (0.29‐1.48)	.303
Treatment		
Intervention on airway stenosis	1	
Excision (pre)malignant lesion	1.88 (0.42‐8.35)	.409
Tumour debulking	2.84 (0.69‐11.75)	.150
Excision Benign lesion	2.76 (0.70‐10.82)	.146
Others or diagnostic procedure	1.11 (0.17‐7.08)	.911
Use of laser		
Yes	1	
No	0.74 (0.28‐1.94)	.544

Note

Statistical test used: univariable logistic regression. Significant *P*‐values are indicated with bold numbers.

Abbreviations: ACE‐27, Adult Comorbidity Evaluation‐27 index; ASA Class, American Society of Anaesthesiologist Physical Status Classification; BMI, Body Mass Index; CI, confidence interval; OR, odds ratio.

### 3.3 Multivariable analysis

Multivariable model, containing BMI, pulmonary pathology, ASA class and percentage of obstruction after backward stepwise elimination, included BMI and pulmonary pathology only (Table 3). In the final model, a 1 kg/m2 higher BMI increased the risk of conversion 1.16 times (95% CI: 1.09‐1.25). Positive history of pulmonary pathology increased the risk 4.91 times (95% CI: 1.93‐12.47).

**Table 3:** Backward stepwise multivariable regression analysis of factors contributing to SSHFJV failure

Variables	OR (95% CI)	*P*‐value
STEP 1		
BMI	1.158 (1.080‐1.242)	**<.001**
Positive history of pulmonary pathology	4.113 (1.512‐11.190)	**.006**
ASA Class of 3 or 4	1.198 (0.436‐3.293)	.726
High percentage of obstruction	1.008 (0.995‐1.022)	.209
STEP 2		
BMI	1.160 (1.082‐1.243)	**<.001**
Positive history of lung pathology	4.352 (1.68‐11.26)	**.002**
High percentage of obstruction	1.009 (1.00‐1.25)	.184
STEP 3		
BMI	1.162 (1.09‐1.25)	**<.001**
Positive history of lung pathology	4.909 (1.934‐12.466)	**.001**

Note

Statistical test used: backward stepwise multivariable logistic regression. Significant *P*‐values are indicated with bold numbers.

Abbreviations: ASA Class, American Society of Anaesthesiologist Physical Status Classification; BMI, body mass index; CI, confidence interval; OR, odds ratio.

## 4 DISCUSSION

### 4.1 Synopsis

This is the first study investigating the predictors of unsuccessful SSHFJV. In this retrospective analysis of 224 adult cases, we confirmed that SSHFJV is applicable in the vast majority of the cases. The risk of conversion to endotracheal intubation is higher in patients with a history of pulmonary disease or elevated BMI. The identification of these predictors may help the surgical team, including surgeons and anaesthesiologists, to prepare for an eventual temporary or definitive conversion to an alternative ventilation technique. An eventual conversion to endotracheal intubation does not jeopardise patient safety if the team is properly prepared. Previous studies focused on the safety, feasibility, limitations, complications of this technique, and analysis of CO_2_‐elimination and gas‐exchange during SSHFJV and not on factors that may influence the success of SSHFJV.^2^, ^3^

### 4.2 Complications during SSHFJV

We found no severe complications, like barotrauma, subcutaneous emphysema, endotracheal fire or death in our series. This is in line with other, larger studies including 500 and 1515 cases.^4^, ^5^

### 4.3 Pulmonary pathology and SSHFJV

We found a significantly increased chance of conversion SSHFJV to endotracheal intubation in patients with a positive history of pulmonary pathology; however, a notable percentage (41/55; 74.5%) of patients with pulmonary pathology could undergo surgery with SSHFJV. In another study, high‐risk patients including patients with COPD, emphysema, bronchial asthma or pulmonary metastases were reported to be adequately ventilated; however, in that series two of three converted cases had pulmonary comorbidities.^5^

### 4.4 Obesity and SSHFJV

Obese patients have impaired oxygen reserve, respiratory mechanics and often diverse comorbidities^7^; therefore, HFJV is expected to be more often difficult. Indeed, we found a higher chance of conversion in patients with a higher BMI. None of the above‐mentioned studies shared that conclusion.^4^, ^5^

### 4.5 Stenosis and SSHFJV

In line with other studies, we experienced no correlation in the multivariable analysis between the severity of the stenosis and the chance of conversion. An Austrian group described safe application of SSHFJV in patients with severe stenosis.^5^ As supraglottic ventilation is applied proximal to the stenosis, it reduces the risk of barotrauma. This is the main advantage of SSHJV compared with jet ventilation with a catheter. Patients with severe stenosis can develop barotrauma when ventilated with a catheter, as the space of gas outflow around the jet catheter can be blocked by the stenosis. Furthermore, ventilation can also be hampered by twisting or kinking of the ventilating catheter during surgical manipulation or it can be obstructed by a mucus plug. In addition, SSHFJV can be used safely in stent application and laser surgery. Another study reported 139 patients with severe laryngeal or tracheal stenosis, and all interventions could be completed without any complications related to the technical ventilation procedure.^4^

### 4.6 CO_2_ laser and SSHFJV

The application of CO_2_ laser requires low oxygen concentration of the ventilating gas in order to avoid airway fire^8^; even though, experts do not unanimously agree on this issue.^4^, ^5^ In our practice, we routinely reduce the O_2_ concentration of the ventilating gas to lower than 40%. Despite this, the application of CO_2_ laser did not increase the chance of conversion from SSHFJV to an alternative ventilation technique. Furthermore, we did not experience any incidents related to CO_2_ laser application during SSHFJV, just like other studies.^4^, ^5^

### 4.7 Strengths and limitations

The study included a consecutive series of patients without any selection; therefore, our database includes high‐risk patients, too. We used validated scoring systems in our analysis which makes our results comparable with other studies. Furthermore, beyond reviewing clinical charts, we have reassessed the clinical photographs in order to minimise missing data and to avoid incorrect data that may come from inaccurate registration.

Of course, the study suffers from its retrospective nature with some missing data and also some bias in the inclusion, as anaesthetists might have contraindicated SSHFJV ahead of the procedure, for instance based on comorbidities. Furthermore, the point of conversion is also strongly depending on the anaesthesiologist: some anaesthesiologists convert earlier, some later.

## 5 CONCLUSIONS

Upper airway surgery ventilated with SSHFJV is possible in the vast majority of the patients. However, clinicians have to be alert in patients with positive history of pulmonary pathology and with higher BMI, as these patients have higher risk for failure.

## CONFLICT OF INTEREST

The authors have no conflict of interest to declare.
